# Summer music and arts festivals as hot spots for measles transmission: experience from England and Wales, June to October 2016

**DOI:** 10.2807/1560-7917.ES.2016.21.44.30390

**Published:** 2016-11-03

**Authors:** Olivier le Polain de Waroux, Vanessa Saliba, Simon Cottrell, Nick Young, Malorie Perry, Antoaneta Bukasa, Mary Ramsay, Kevin Brown, Gayatri Amirthalingam

**Affiliations:** 1 National Infection Service, Public Health England, London, United Kingdom; 2 Immunisation, Hepatitis and Blood Safety Department, National Infection Service, Public Health England, London, United Kingdom; 3 Communicable Disease Surveillance Centre and Vaccine Preventable Disease Programme, Public Health Wales, Cardiff, United Kingdom; 4 Public Health England South West, Public Health England, Exeter, United Kingdom; 5 Virus Reference Department, National Infection Service, Public Health England, London, United Kingdom

**Keywords:** Europe, United Kingdom, airborne infections, viral infections, measles, mass gatherings, measles-mumps-rubella (MMR) vaccine, outbreaks, vaccines and immunisation, epidemiology

## Abstract

We report 52 cases of measles linked to music and arts festivals in England and Wales, between mid-June and mid-October 2016. Nearly half were aged 15 to 19 years. Several individuals who acquired measles at one festival subsequently attended another festival while infectious, resulting in multiple interlinked outbreaks. Transmission within festivals resulted in a geographical spread of cases nationally as well as internationally, which presents particular challenges for measles control.

Since the start of summer 2016, increasing numbers of individuals with measles who attended music and arts festivals have been reported in England and Wales. These have occurred in the context of an ongoing measles outbreak which began earlier in the year [1], and predominantly affected adolescents and young adults (age: 14–40 years) in London and East of England [2]. Here we describe 52 measles cases that were identified between mid-June and mid-October 2016.

## Case definitions and inclusion criteria for the study

Any suspected case of measles, defined as a person presenting with clinically compatible symptoms, which was reported to a health protection team (HPT) between 15 June and 15 October was initially considered for inclusion in the study. For the analysis, we selected only confirmed cases based on detection of measles specific IgM in serum or oral fluid and/or measles RNA detection in either a local laboratory or the reference laboratory, as well as epidemiologically-confirmed cases, which were suspected cases epidemiologically linked to another confirmed case of measles [3]. Cases included in the analysis were further restricted to those, which were either infectious at a festival (i.e. index cases) or which developed measles within an incubation period of their festival attendance (i.e. secondary cases). We did not include cases indirectly linked to festivals through tertiary (or further) transmission chains. Any public mass gathering labelled as a festival (whether of arts or music or both) was considered for this report. HPTs managing each measles case entered festivals as a specific context on HPZone or IBID, the respective web-based case management systems of Public Health England (PHE) and Public Health Wales (PHW) that such teams locally use.

We identified index cases based on their dates of onset of rash, and dates of the festival(s) attended, assuming a period of infectiousness from four days before to four days after rash onset [4]. Individuals who developed symptoms within the minimum and maximum known incubation period for measles from first (minimum) and last (maximum) day of the festival attended were considered secondary cases, assuming a range of 7 to 21 days from infection to prodromal symptoms [4], and another 2 to 4 days before the rash appears [4]. 

## Data retrieval

Epidemiological and clinical details on measles cases from England and Wales were obtained from HPZone for cases under the management of PHE or from IBID for cases under the management of PHW. Data on confirmatory testing, genotyping and further strain characterisation were obtained from the United Kingdom (UK) national reference laboratory at PHE. 

## Description of the festival-related outbreak

As of 15 October 2016 a total of 56 cases potentially linked to festivals in England and Wales have been reported. Information about the festivals attended and dates of attendance was unavailable for four cases, leaving 52 cases for analysis. Of those, most (n=47) were confirmed cases and the remaining five were epidemiologically-confirmed cases. Overall, the median age of cases was 19 years (range: 11 months–52 years) with almost half (n=24) being between 15 and 19 years-old. A total of 23 cases were female (Table). Most cases (n=42) were unvaccinated, four reported having had a single dose of a measles-containing vaccine, one confirmed case was fully vaccinated and the vaccination status of five other confirmed cases was unknown. Twenty-one cases were admitted to hospital (defined as being admitted for at least one night), and there were no deaths among such cases. The hospitalisation rate increased with age, from eight among the 30 individuals less than 20 year-olds to 12 among the 21 aged 20 years and above (p = 0.043). Further details are provided in the Table.

**Table:** Characteristics of measles cases and the festivals that they attended, England and Wales, 15 June–15 October 2016 (n=52 cases)

Characteristics of cases	Number
**Age in years**	
< 10	3
10–14	4
15–19	24
20–24	12
≥25	9
**Sex**	
Male	29
Female	23
**Vaccination status**	
Unvaccinated	42
1 dose of measles-containing vaccine	4
2 doses of measles-containing vaccine	1
Vaccination status unknown	5
**Hospitalisation^a^**	
No	30
Yes	21
Not known	1
**Festival name (type)**	**Approximate size**
Triplicity Music and Arts festival (music and arts)	1,000–2,000
Glastonbury festival (music)	144,000
NASS festival (music and sports, including skateboarding and BMX)	4,000–5,000
Workhouse festival (music, arts and crafts)	500–1,000
Tewkesbury Medieval festival (medieval festival, re-enacting the battle of Tewkesbury)	25,000
Noisily festival (music)	3,000
Nozstock festival (music)	5,000
Secret Garden Party festival (music)	25,000
Green Gathering festival (music and arts)	4,000–5,000
Boomtown music festival (music)	60,000
Beautiful Days festival (music)	16,500
Shambala music festival (music and arts)	7,000

BMX: bicycle motocross.

^a ^Admitted to hospital for at least one night.

**Figure 1:**
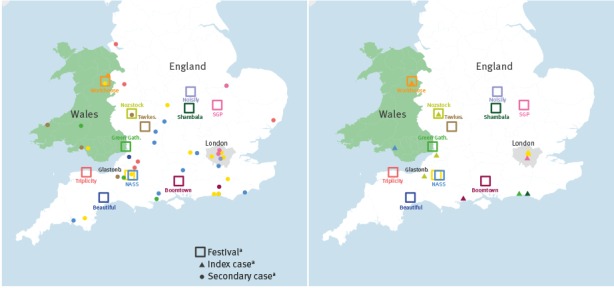
Geographical distribution of index measles cases (right panel), secondary cases (left panel), and festivals (both panels) in England and Wales, 15 June–15 October 2016 (n=52 measles cases)

Cases were linked to twelve different festivals, which took place between mid-June and late August in various parts of England and Wales, including large mainstream music festivals such as Glastonbury, as well as smaller independent music and arts festivals (Table and Figure 1).

Beautiful: Beautiful Days festival; Boomtown: Boomtown music festival; Glastonb.: Glastonbury festival; Green Gath.: Green Gathering festival; NASS: NASS festival; Noisily: Noisily festival; Nozstock: Nozstock festival; SGP: Secret Garden Party festival; Shambala: Shambala music festival; Tewkes.: Tewkesbury Medieval festival; Triplicity: Triplicity Music and Arts festival; Workhouse: Workhouse festival.

The squares and the dots are centred around the centroid of the postcode district (i.e. the centroid of the postcode district of residence of the cases, or the centroid of the postcode district of the locations of the festival).

^a^ Each festival is indicated by a square of a different colour. The colours of triangles (index cases) and dots (secondary cases) correspond to the festivals that the cases were linked to. The maps do not include one Scottish resident linked to Noisily festival (secondary case).

Although we were unable to obtain detailed denominator data for each festival, available estimates suggest that the attack rate remained low, < 1 per 1,000 in most festivals, ranging from approximatively 0–6 per 1,000 individuals. Eight cases were identified as working or performing at the festival.

**Figure 2:**
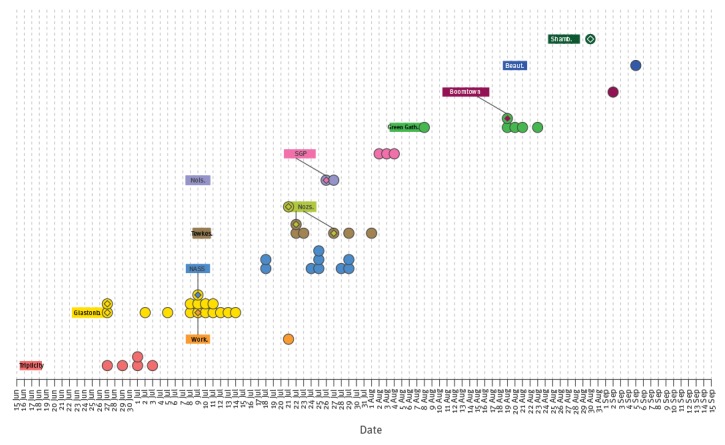
Date of onset of rash for each measles case, in relation to the timeline of the festival(s) cases were linked to, England and Wales, 15 June–15 October 2016 (n=52 cases)

One or more index cases could be identified in eight of the twelve festivals respectively (Figures 1 and 2). Six individuals who acquired their infection at one festival subsequently attended another festival while unwell (Figure 2), resulting in multiple interlinked outbreaks, and four of them were festival performers or workers. One of the workers is known to have attended the festival for only a few hours, and left due to illness, but all other index cases (including non-workers) are not known to have left the festival because of illness. The proportion of hospitalised index cases was similar to that of secondary cases (p = 0.355), suggesting no difference in severity.

Beaut.: Beautiful Days festival; Boomtown: Boomtown music festival; Glastonb.: Glastonbury festival; Green Gath.: Green Gathering festival; NASS: NASS festival; Nois.: Noisily festival; Nozs.: Nozstock festival; SGP: Secret Garden Party festival; Shamb.: Shambala music festival; Tewkes.: Tewkesbury Medieval festival; Triplicity: Triplicity Music and Arts festival; Work.: Workhouse festival.

The length of each rectangle represents the duration of the festival. Each circle corresponds to a case, and the date against which they are shown is the date of onset of rash. The colour of each circle corresponds to the colour of the festival from which cases most likely acquired measles. Diamond shapes are shown within circles for index cases, and the colour of the diamond corresponds to the festival for which cases are index cases. Lines between circles and festivals are shown for individuals who have attended multiple festivals.

The Glastonbury outbreak commenced with the attendance at the festival of two different index cases, both London residents. Of the 14 secondary cases in England and Wales, one later attended the NASS festival while symptomatic, and another was symptomatic at Workhouse festival, which resulted in secondary transmission in both festivals (Figures 1 and 2). Eight samples from Glastonbury cases and one from a secondary case linked to NASS were genotyped, and all were genetically identical to the strain circulating in London and East of England since February 2016 (MVs/Cambridge.GBR/5.16/[D8]; GenBank: KX161662). The findings are therefore consistent with an initial introduction at Glastonbury from London, and subsequent spread to other festivals. Similarly, although no index case was identified for Tewkesbury festival, the same ‘Cambridge-D8’ strain was found in the only sample genotyped. In contrast, cases linked to the Triplicity festival were of a distinct genotype D8 strain (MVs/Torquay.GBR/22.16/3 [D8]; GenBank: KX757048), which had only been observed in the South West region where the festival was held, suggesting introduction from a local resident.

Cases associated with festivals spread to areas where few or no cases had previously been notified, including Wales (7 cases) and Scotland (1 case).

Three cases linked to three different festivals later travelled to European countries during their period of infectiousness (and sometimes illness), including to France, Germany and Spain. The early warning and response system (EWRS) was used to exchange information about these cases with the respective European Union Member States.

We are also aware of another secondary case not included in our analysis, among an adult foreign resident linked to Glastonbury festival who became unwell in Germany, and infected a tertiary case whose genotyping confirmed the same strain than other Glastonbury cases (Anne Belting, personal communication, August 2016).

## Discussion

Music and arts festivals present the ideal environment for the transmission of infections like measles, given the large crowds and close prolonged interpersonal contact between individuals [5,6]. The popularity of these events [7] enhances the potential for outbreaks in this type of setting, and it is therefore important to advise festival goers, as well as individuals working or performing at such events, to be fully vaccinated.

Although relatively small and contained measles outbreaks have occurred in UK festivals in the past [8] transmission between festivals within the country has not been previously described. While the attack rates in each of the eight festivals described in this report remained low, transmission from a single introduction could lead to up to nine secondary cases, as in the NASS festival. Taking into account the levels of population immunity to measles, this suggests that the number of effective contacts for measles in festival settings may be particularly high.

Our data indicate that measles could spread from one festival to another, mostly through symptomatic festival workers or artists on festival tours. However, we also show in this report that transmission occurs through festival goers themselves, 60% of whom will attend more than one festival during the summer period in the UK [7]. Festival-related measles outbreaks may also spread internationally, particularly given that an estimated 3% of festival attendees in the UK are originating from abroad [7]. Because many festival attendees from the UK are also likely travel abroad during the summer period, transmission in the UK can have implications for other countries. This is highlighted in our recent experience with secondary cases in other European countries and presents particular challenges for measles control.

Interestingly, more than half of the cases aged ≥ 15 years were between 15 and 19 years of age. While a precise age breakdown of festival goers is not known, the average age of festival goers is increasing, and is now around 35 years of age in the UK [7], thereby suggesting that teenage attendees might have been disproportionally affected. This likely reflects the higher susceptibility in that age group, the 15 to 19 year-olds belong to birth cohorts in whom preschool measles, mumps, and rubella (MMR) vaccine coverage was low in the late 1990s and early 2000s (76% MMR coverage at 5 years in 1999/2000 [9,10], 74% at 5 years in 2005/2006 [11]) and who were targeted in 2013 by a national catch-up campaign [10,12,13]. However, despite this, a proportion of teenagers from these birth cohorts remains unvaccinated [13], and may therefore be more prone to measles (and mumps) as they now start attending festivals. Further research, including targeted seroprevalence surveys, may be useful to explore this further. 

Our analysis was based on cases that were reported as linked to festivals and so some cases will have been missed, including the index cases from three festivals. This may be due to festival exposure not being recorded, but more likely reflects some cases not presenting for healthcare or not being diagnosed as measles. The relatively high rate of hospitalisation in our case series may be age-related, but could also reflect a reporting bias towards more severe cases, if milder cases do not seek care. The fact that several cases attended the festivals during their illness suggests some infections were relatively mild among some festival goers.

To control the outbreaks, PHE and PHW issued communications, warning festival goers about measles, advising them to ensure that they were fully vaccinated, and urging staff, artists and festival goers not to attend festivals if unwell [1,14]. These measures are also important, for any other type of mass gathering event.
